# Assessing body image in anorexia nervosa using biometric self-avatars in virtual reality: Attitudinal components rather than visual body size estimation are distorted

**DOI:** 10.1017/S0033291717002008

**Published:** 2017-07-26

**Authors:** S. C. Mölbert, A. Thaler, B. J. Mohler, S. Streuber, J. Romero, M. J. Black, S. Zipfel, H.-O. Karnath, K. E. Giel

**Affiliations:** 1 Department of Psychosomatic Medicine and Psychotherapy, Medical University Hospital Tübingen, Tübingen, Germany; 2 Max Planck Institute for Biological Cybernetics, Tübingen, Germany; 3 Graduate Training Centre of Neuroscience, International Max Planck Research School, Universität Tübingen, Tübingen, Germany; 4 École Polytechnique Fédérale de Lausanne, Brain Mind Institute, Lausanne, Switzerland; 5 Max Planck Institute for Intelligent Systems, Tübingen, Germany; 6 Division of Neuropsychology, Center of Neurology, Hertie-Institute for Clinical Brain Research, University of Tübingen, Tübingen, Germany

**Keywords:** Anorexia nervosa, body image disturbance, body size estimation, eating disorders

## Abstract

**Background:**

Body image disturbance (BID) is a core symptom of anorexia nervosa (AN), but as yet distinctive features of BID are unknown. The present study aimed at disentangling perceptual and attitudinal components of BID in AN.

**Methods:**

We investigated *n* = 24 women with AN and *n* = 24 controls. Based on a three-dimensional (3D) body scan, we created realistic virtual 3D bodies (avatars) for each participant that were varied through a range of ±20% of the participants’ weights. Avatars were presented in a virtual reality mirror scenario. Using different psychophysical tasks, participants identified and adjusted their actual and their desired body weight. To test for general perceptual biases in estimating body weight, a second experiment investigated perception of weight and shape matched avatars with another identity.

**Results:**

Women with AN and controls underestimated their weight, with a trend that women with AN underestimated more. The average desired body of controls had normal weight while the average desired weight of women with AN corresponded to extreme AN (DSM-5). Correlation analyses revealed that desired body weight, but not accuracy of weight estimation, was associated with eating disorder symptoms. In the second experiment, both groups estimated accurately while the most attractive body was similar to Experiment 1.

**Conclusions:**

Our results contradict the widespread assumption that patients with AN overestimate their body weight due to visual distortions. Rather, they illustrate that BID might be driven by distorted attitudes with regard to the desired body. Clinical interventions should aim at helping patients with AN to change their desired weight.

Anorexia nervosa (AN) is a serious eating disorder that goes along with high rates of psychological and physical comorbidity as well as with increased levels of disability and mortality (Zipfel *et al.* 2015; Fichter & Quadflieg, 2016). Treatment of AN is expensive and often yields sub-clinical symptoms rather than complete remission (Egger *et al.* 2016; Schmidt *et al.* 2016). In addition to self-induced underweight and circumvention or even fear of gaining weight, body image disturbance (BID) is a core symptom of AN (American Psychiatric Association, 2013). As yet, distinctive features and mechanisms of BID remain unclear, specifically in regards to the contributions of sensory perceptual distortions *v*. cognitive–affective disturbance (Dakanalis *et al.* 2016; e.g. Frank & Treasure, 2016). To improve the clinical treatment of AN, a deeper understanding of BID in AN is needed.

There is consistent evidence that cognitive–affective components of body image are disturbed in AN. Several studies found that patients with AN report higher body dissatisfaction, weight and shape concerns, higher drive for thinness and a thinner desired weight than control participants (Cash & Deagle, 1997; Zipfel *et al.* 2014; Moscone *et al.* 2017). Other studies observed that patients with AN are satisfied with their weight (Striegel-Moore *et al.* 2004; Benninghoven *et al.* 2007), which given their underweight is interpreted to reflect a disturbed body image, as well. It has been repeatedly suggested that sensory perceptual distortions might underlie these findings in the sense that patients with anorexia nervosa (AN) ‘see’ their bodies fatter than they really are or that they do not recognize weight loss (Bruch, 1962; Slade & Russell, 1973; Farrell *et al.* 2005).

Indeed, several studies observed that patients with AN overestimate their body size in different visual size estimation tasks (Mölbert *et al.* n.d.; Cash & Deagle, 1997; Farrell *et al.* 2005; Gardner & Brown, 2014) and even in non-visual measures (Gaudio *et al.* 2014). However, the interpretation of the overestimation effect as indicative for perceptual distortion has been questioned: The magnitude of overestimation has been found to be sensitive to the instruction wording such that a focus on ‘knowledge’ *v*. ‘feelings’ often reduced or even revoked the overestimation (Proctor & Morley, 1986; Bowden *et al.* 1989; Caspi *et al.* 2017). Additionally, it has been suggested that demand characteristics influenced performance, as patients with AN might have thought they were asked to illustrate their experience of being ‘too fat’ (Smeets, 1997). This explanation is supported by psychophysics studies that did not replicate overestimation (Gardner & Moncrieff, 1988; Gardner & Bokenkamp, 1996; Smeets *et al.* 2009).

An alternative explanation suggests that overestimation might be a secondary effect of the low weight of individuals with AN, as a contraction bias could distort their estimates toward the average body weight (Cornelissen *et al.* 2013, 2015). This explanation, however, implies that patients with AN should also overestimate the weight of other thin people. Interestingly, some recent studies observed that patients with AN indeed tend to overestimate other people's weight when rating their weight in categories (Horndasch *et al.* 2015; Moody *et al.* 2016). In contrast, another study observed that participants with AN accurately memorized and adjusted another person's body (Øverås *et al.* 2014). Hence, it is still unclear under which circumstances patients with AN overestimate weight and how this overestimation is characterized.

In this study, we made use of recent technical advances to assess the contributions of both cognitive–affective and perceptual processes to the body weight estimation in AN. Specifically, we used a stereoscopic virtual reality life-size stereo display, a three-dimensional (3D) body scanner and a statistical body model that allow for realistic weight manipulations of photo-realistic virtual avatars and naturalistic mirror-scenario presentation of these avatars. Importantly, this technology also enabled us to create artificial other persons that had the participant's body shape and weight. To reduce demand characteristics, we used psychophysical tasks and an outside-treatment-setting, and investigated the following questions: (1) Do women with AN overestimate their weight or do they differ in their sensitivity to weight change as compared to controls? (2) How do women with AN differ from controls with regard to their desired body? (3) Are estimated own body size or desired body size correlated with eating disorder symptoms or own body weight? Further, to investigate the influence of a low body weight on perception of other persons’ weight in AN, we conducted a second experiment asking. (4) Do size estimates and most attractive body weight change when they refer no longer to the own body but to another person who is matched in body shape and weight? Finally, we also invited participants back for a replication of Experiment 1 in 2D to find out (5) How robust are our findings on own body size estimation and desired body size?

## Methods

### Participants

*n* = 24 women with AN diagnosed according to DSM-5 and *n* = 24 age and gender matched normal weight control participants with no history of mental disorders gave their informed written consent and participated in the study. Exclusion criteria for all participants were current pregnancy or lactation, diseases of the central nervous system, alcohol- or drug dependence, schizophrenia, bipolar disorder, and acute suicidal tendency. Women with AN were recruited from the inpatient (*n* = 23) and outpatient (*n* = 1) service of the Department of Psychosomatic Medicine and Psychotherapy at the University Hospital Tübingen. The experimenter was not part of the therapeutic team, and women with AN were informed that data assessed in the study would not be shared with the therapeutic team. At study inclusion, patients in inpatient treatment were treated for Md = 4 weeks (Min = 1 week, Max = 16 weeks). The study was approved by the local ethics committee of the University Tübingen and the Medical Faculty Tübingen.

### Stimulus generation and technical setup

**Fig. 1.**
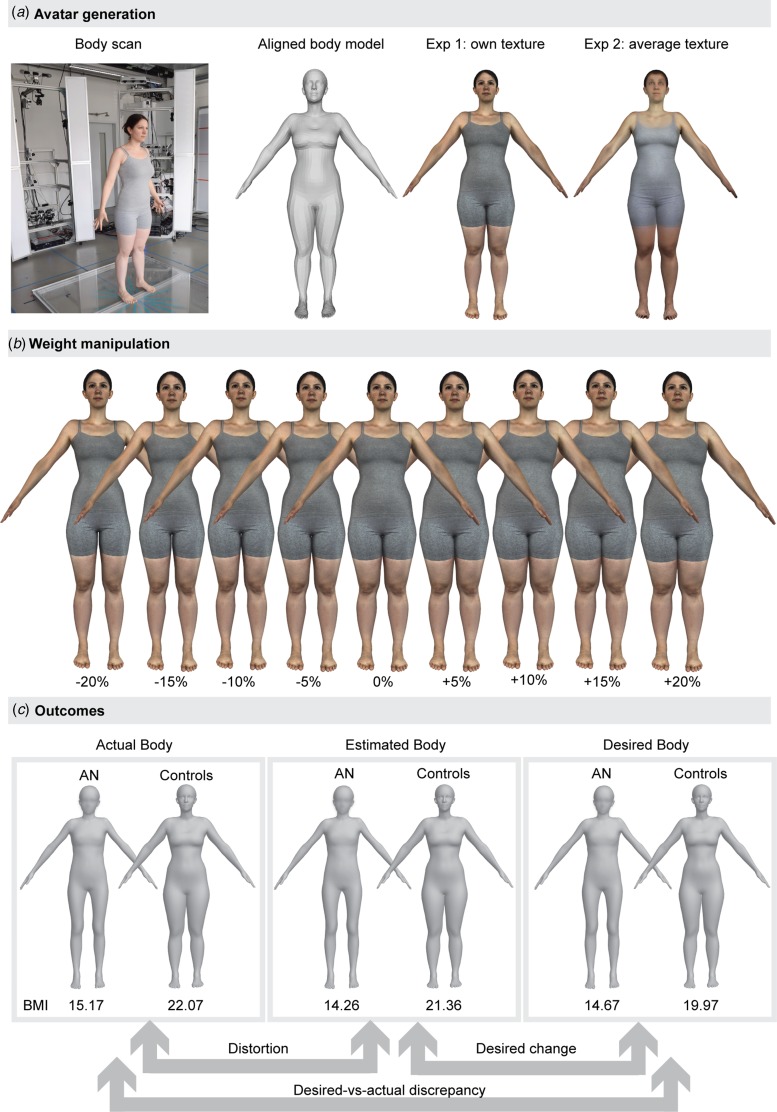
(*a*) Avatar generation based on a 3D body scan for Experiment 1 (own photo-realistic texture) and Experiment 2 (average texture). (*b*) Illustration of weight manipulations. (*c*). Illustration of outcome parameters, average actual and average adjusted body mass index (BMI; kg/m^2^) in Experiment 1 (Method of Adjustment task). The depicted persons provided written consent to be shown in publications.

For each participant, we generated two individual avatars: For Experiments 1 and 3, a 3D photo-realistic self-avatar that could be morphed in a range of ±20% weight and for Experiment 2, a 3D avatar that was matched in weight and body shape, could also be morphed in the range of ±20% weight, but had another identity (Fig. 1*a*). To record the participant's body shape and to generate the individual photo-realistic appearance (texture), we used a full-body scanning system (3dMD, Atlanta/GA). The body shape data was afterwards registered to a parametric model of body shape (Anguelov *et al.* 2005; Hirshberg *et al.* 2012). For each participant, the individual parametrized body shape was then distorted based on weight-associated shape deformations found in the 2094 women from the CAESAR dataset of body scans (Robinette *et al.* 1999) to reflect weight changes of±20% (Fig. 1*b*). The weight and shape matched avatars for Experiment 2 were generated by keeping the individual avatar's original geometry (same height, weight and exact body shape) while replacing its texture with a standard appearance [predefined eye and hair color, clothes, etc., cf. Piryankova *et al.* (2014)].

In Experiments 1 and 2, avatars were presented on an immersive life-size stereoscopic display that mimicked in virtual reality the situation of looking at oneself in a mirror. In Experiment 3, avatars were presented in 2D on an ordinary desktop monitor. A detailed description of the stimulus generation and technical setup is provided in the supplement.

### Procedure

The procedure comprised: (1) A diagnostic session (1–2 h), (2) the 3D body scan (20 min), (3) an experimental session with Experiments 1 and 2 (1 h) and optionally (4) Experiment 3, a desktop replication of Experiment 1 at least 1 week later (30 min). Session 1–3 took place within 3–17 days. In the diagnostic session, the eating disorder examination interview on eating behavior, attitudes toward weight and shape (EDE; Cooper & Fairburn, 1987; Hilbert & Tuschen-Caffier, 2010), and the SCID-I interview parts on affective disorders, substance abuse, anxiety disorders, and somatoform disorders (Wittchen *et al.* 1997) were conducted. Further, questionnaires were administered to assess self-esteem (Rosenberg SES; Rosenberg, 1965; Ferring & Filipp, 1996; von Collani & Herzberg, 2003), body image (FKB-20; Clement & Löwe, 1996; EDI-2 scales ‘Drive for Thinness’ and ‘Body Dissatisfaction’; Paul & Thiel, 2005) and social comparison tendencies (PACS; Mölbert *et al.* 2017).

At the beginning of the experimental session, each participant was informed that based on her body scan, an exact model and more or less manipulated models of her body had been generated. Manipulations were explained using a balloon analogy stating that an algorithm had manipulated the participant's body as if one would blow up or shrink a balloon. The participant was told that she would now see different versions of her body and had to decide whether the version was exactly her body or a manipulated version. In Experiment 1, participants estimated the size of their own body with photo-realistic texture and indicated their desired body size. In Experiment 2, participants estimated the size of the weight and shape matched avatar that they memorized before. All instructions were then modified to refer to the memorized avatar. Experiment 3 followed the procedure of Experiment 1.

Each experiment consisted of three tasks: In the One-Alternative-Forced-Choice (1AFC) task, participants randomly saw bodies at ±0, 5, 10, 15, and 20% of their weight each 20 times for 2 s and afterwards had to indicate whether they agreed to the statement ‘This is my body’ or not (in case they thought it was a manipulated version). In Experiment 2, the statement was modified to ‘This is the correct body’. In the Method of Adjustment (MoA) tasks, participants could continuously adjust the avatar in steps of 0.05% of participant's body mass index (BMI) within the ±20% weight range, and were instructed to adjust it nine times to their current and nine times to their ideal weight. Each of the nine avatars from the 1AFC task was used as a starting avatar once. In Experiment 2, the instructions referred to the remembered or the most attractive body. The order of the experiments and tasks were kept constant to keep participants as naïve as possible for Experiment 1. Before and after the experimental session, participants filled out the state-trait-anxiety questionnaire in its state form (Laux *et al.* 1981). Further, after the session, participants completed a post-questionnaire asking to rate on a Likert scale from 1 (not at all) to 7 (very much) how similar they perceived the two avatars (overall impression) to themselves and whether they identified with their avatar. Piryankova *et al*. (2014) observed such ratings to be sensitive to dissimilarities between avatar and participant. A more detailed description of the experimental procedure is provided in the supplement.

## Results

### Sample

Sample characteristics are summarized in Table 1. Participants with AN and controls did not differ with respect to age, but in terms of height, weight, BMI, body dissatisfaction, self-esteem, comparison habits with regard to outer appearance and eating disorder symptoms (Table 1). 30% of women with AN fulfilled DSM-5 criteria for comorbid major depression. Women with AN reported that they had received the diagnosis of AN for the first time Md = 3 years ago (Min = 0 years, Max = 23 years). According to DSM-5 severity classification, 21/24 (87.5%) women with AN had extreme AN in the past, 2/24 (8.3%) had severe AN in the past and one (4.1%) had moderate AN in the past. At study intake, AN was classified as extreme in 13/24 (54.1%), as severe in 3/24 (12.5%), as moderate in 5/24 (20.8%) and as mild in 3/24 (12.5%) of women with AN. Women with AN had higher levels of anxiety before the experimental session (3) but reduced their anxiety throughout the session as much as controls did (Table 1). Due to organizational and technical issues, missing data occurred in most of the assessed variables and outcome parameters, but it was randomly distributed and affected only 2.7% of all values over the whole sample from analyzed data, so that we opted against imputation.

**Table 1.** Sample characteristics and group comparisons (t tests and effect size d) for age, body mass index, interview and questionnaire data

	Women with AN					Controls						
	M	s.d.	Md	Min	Max	M	s.d.	Md	Min	Max	Sig.	*d*
Age	24.00	6.35	21.00	19.00	39.00	24.13	6.42	21.00	18.00	41.00	n.s.	0.01
BMI (kg/m^2^)	15.17	1.47	14.97	12.68	17.96	22.07	1.85	21.50	19.41	25.51	***	2.08
EDE Total	2.23	1.05	2.43	0.51	4.38	0.33	0.28	0.29	0.00	0.96	***	1.42
EDE Res	2.53	1.40	2.80	0.00	4.60	0.34	0.62	0.00	0.00	2.20	***	1.08
EDE EC	1.45	1.07	1.40	0.00	3.80	0.04	0.10	0.00	0.00	0.40	***	1.20
EDE WC	2.42	1.41	2.00	0.40	6.00	0.46	0.56	0.30	0.00	2.20	***	1.00
EDE SC	2.52	1.30	2.00	0.63	5.13	0.49	0.29	0.50	0.00	1.00	***	1.27
EDI-2- DT	29.04	8.11	31.50	9.00	40.00	12.92	6.44	10.50	7.00	32.00	***	1.11
EDI-2-BD	35.50	8.93	37.00	14.00	54.00	23.75	8.99	25.00	9.00	50.00	***	0.66
BIQ-VBD	27.56	6.01	30.00	15.00	39.00	37.38	5.32	37.50	27.00	45.00	***	0.87
BIQ-NBE	33.58	8.66	33.50	17.00	50.00	17.71	5.23	16.00	11.00	38.00	***	1.14
RSE	13.08	7.00	12.50	3.00	27.00	24.17	4.67	24.50	11.00	30.00	***	0.95
PACS	17.50	3.15	17.00	12.00	25.00	11.88	3.98	12.00	5.00	23.00	***	0.78
STAI pre	47.67	10.33	47.00	30.00	70.00	31.83	6.07	31.50	21.00	49.00	***	0.97
STAI Diff.	−1.77	10.22	−2.00	−19.00	31.00	−0.17	4.69	−2.00	−5.00	17.00	n.s.	0.11

***p < 001 after Bonferroni-correction.

BMI, body mass index; EDE, eating disorder examination interview (Cooper & Fairburn, 1987; Hilbert & Tuschen-Caffier, 2010); EDE Total, EDE total score; EDE Res, subscale restraint, EDE EC, subscale eating concerns; EDE WC, subscale weight concerns; EDE SC, subscale shape concerns; EDI-2, Eating Disorder Inventory – 2 (Paul & Thiel, 2005); EDI-2-DT, subscale Drive for Thinness; EDI-2-BD, Subscale Body Dissatisfaction; BIQ, Body Image Questionnaire FKB-20 (Clement & Löwe, 1996); BIQ-VBE, subscale vital body dynamics; BIQ-NBE, subscale negative body evaluation; RSE, Rosenberg Self Esteem Scale (Ferring & Filipp, 1996; von Collani & Herzberg, 2003); PACS, Physical Appearance Comparison Scale (Thompson *et al.* 1991; Mölbert *et al.* 2017); STAI, State Trait Anxiety Inventory; State-Form (Laux *et al.* 1981); STAI pre, before experiment; STAI Diff, change after experiment.

### Manipulation check

In the post-questionnaire, 75% of participants in each group stated that they felt the avatar represented themselves in virtual reality. All participants stated that they experienced the avatar with own appearance in Experiment 1 as more similar to themselves than the weight and shape matched avatar with another identity in Experiment 2 [women with AN: mean *self* = 5.59 (s.d. = 0.96), *other* = 4.18 (1.62); Controls: *self* = 6.25 (1.03), *other* = 4.88 (1.57); *F*_(1,44)_ = 30.36, *p* < 0.001]. Women with AN, however, generally rated the avatars as less similar to themselves than controls [*F*_(1,44)_ = 5.01, *p* *<* *0*.05].

### Experiment 1: Perception of own body weight

**Fig. 2.**
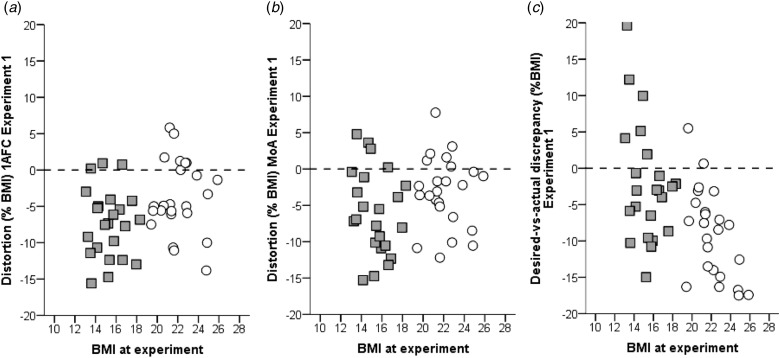
Distortion as measured by the One-Alternative-Forced-Choice task (*a*) and the Method-of-Adjustment task (*b*) and Desired-*v*.-actual Discrepancy (*c*) in percent of participants’ actual weight in Experiment 1 (own photo-realistic texture) depending on personal BMI of the participants. Gray squares: Women with AN. White circles: Controls. The dashed horizontal line indicates hypothetical accurate performance/no desire for weight change. Positive values reflect overestimation/ a higher desired than actual body weight, negative values reflect underestimation/a lower desired than actual weight.

Figure 1*c* and Table 2 provide an overview on the experimental outcome parameters for both groups. Details on the parameter calculation and the statistical analysis are provided in the supplement. *T* tests were used to test whether the parameters significantly differed from zero; group differences were analyzed with univariate analyses of variance (ANOVAs). The outcome parameter *distortion*, reflecting the over- or underestimation in terms of percent of individual actual body weight, was negative and significantly different from zero in both groups and tasks, indicating that both groups consistently underestimated their actual body size in both the 1AFC task and the MoA task (Fig. 2). According to the *distortion* parameter derived from the 1AFC task, women with AN underestimated their weight even more than women in the control group [*F*_(1,45)_ = 6.35, *p* < 0.05]. However, for the distortion parameter derived from the MoA task there was only a trend towards a group difference [*F*_(1,45)_ = 3.09, *p* = 0.086].

**Table 2.** Means (M), Standard Deviations (s.d.) and group comparisons (F Test and effect size Eta^2^) for outcome parameters of Experiment 1

	Women with AN (*n* = 23)		Controls (*n* = 24)		sig	*η* ^2^
	M	s.d.	M	s.d.		
Distortion 1AFC	−7.38	4.71	−3.80	5.02	*	0.12
Distortion MoA	−5.94	5.81	−3.19	4.89	†	0.06
Sensitivity to weight loss^a^	1.14	0.86	1.54	0.79	n.s.	0.06
Sensitivity to weight gain^a^	1.76	0.46	1.77	0.64	n.s.	0.00
Desired change (MoA)	+2.85	8.28	−6.05	4.33	***	0.32
Desired-*v*.-actual discrepancy	−2.11	8.12	−9.08	6.13	**	0.20

†*p* < 0.10, *p < 0.05, **p < 0.01, ***p < 0.001. Only ** and *** would survive correction for multiple testing. All parameters except for Desired change and Desired-*v*.-actual discrepancy in the AN group were significantly different from zero with *p* < 0.001 (one-sample *t* test). Distortion: discrepancy between estimated current and actual body in percent of actual weight, Sensitivity to weight loss: ln-transformed beta values of Weibull fitted 1AFC data left from peak, Sensitivity to weight gain: ln-transformed beta values of Weibull fitted 1AFC data right from peak. Lower beta values reflect lower sensitivity, i.e. a greater tendency to accept the weight manipulated avatars as equal to the actual weight. Desired change: Difference between desired and estimated weight in percent of actual weight. Desired-*v*.-actual discrepancy: Discrepancy between desired body and actual body in percent weight.

a Sample size *n* = 21 AN/*n* = 23 Controls.

*Sensitivity* to weight changes was parametrized in the beta-values from fitting cumulative Weibull functions to the left and right side of the peak of the 1AFC answer distributions (Wichmann & Hill, 2001). High beta values reflect steep slopes and therefore high sensitivity to weight changes. *Sensitivity* was lower for changes in the direction of weight loss than for changes in the direction of weight gain relative to own estimated body weight [*F*_(1,42)_ = 6.77, *p* < 0.05], indicating that participants were more willing to accept a thinner body as their own than a fatter body. Importantly, *sensitivity* did not differ between women with AN and controls: Neither the main effect of group [*F*_(1,42)_ = 2.01, *p* = 0.13] nor the interaction side by group [*F*_(1,42)_ = 1.52, *p* = 0.22] was significant.

*Desired change* of weight, defined as percent weight difference between the estimated and desired body, was significantly different from zero only in the control group (Table 2). Interestingly, 14 women with AN (61%) but only one (4%) control indicated they wanted to gain weight. Consequently, the average *desired change* differed significantly between groups [*F*_(1,45)_ = 21.63, *p* *<* 0.001]. Of note, in twelve of the 14 women with AN indicating they wanted to gain weight, this was in the range of 1–10% of their estimated (and in fact significantly underestimated) weight.

*Desired-v.-actual discrepancy*, reflecting the discrepancy between desired and actual body, was negative and significantly different from zero only in the control group (Table 2). Although 14 women with AN desired to gain weight, only six (26%) actually adjusted their avatar to a weight that was higher than their actual current weight (Fig. 2). On average, the desired body was weighing less than the actual body in both groups, although even more so in the control group [*F*_(1,45)_ = 11.10, *p* *<* 01]. The average desired body of the control group still had a BMI of 19.97 and thus was in normal weight range, while the average desired body of women with AN had a BMI of 14.67, which would correspond to extreme AN in DSM-5 (Fig. 1).

The group wise correlations between experimental parameters for the self-texture condition, BMI and questionnaires are provided in Table 3. The correlation analysis revealed that the only consistent pattern emerged between body dissatisfaction-related parameters, as reflected by the correlations of *desired change* and *desired-v.-actual discrepancy* with BMI, questionnaire measures of body dissatisfaction, restrictive eating, self-esteem and the amount of body-related comparisons. This pattern was more consistent in the control group than in the women with AN. We further observed correlations with questionnaire and EDE interview scores for *distortion* (*MoA*) and *sensitivity* to weight loss, but they were not consistent and only present in the control group. None of the correlations survived Bonferroni correction for multiple testing.

**Table 3.** Pearson correlations of outcome measures with body mass index (BMI), eating pathology, body dissatisfaction, self esteem, comparison behavior and anxiety before the experiment.

	Distortion (1AFC)		Distortion (MoA)		Sensitivity to Weight Loss		Sensitivity to Weight Gain		Desired change (MoA)		Desired-*v*.-actual discrepancy	
	AN	Con	AN	Con	AN	Con	AN	Con	AN	Con	AN	Con
BMI (kg/m^2^)	0.02	−0.01	−0.22	−0.04	0.02	0.16	0.15	0.43	−0.24	−0.67**	−0.44*	−0.59**
EDE Total	0.09	−0.19	0.12	−0.41*	0.23	0.42	0.09	0.08	−0.27	−0.46*	−0.24	−0.63**
EDE Res	−0.26	−0.07	−0.14	−0.18	0.25	0.37	−0.17	0.10	−0.20	−0.51*	−0.34	−0.48*
EDE WC	0.27	−0.32	0.26	−0.49*	0.13	0.26	0.15	−0.15	−0.13	−0.17	0.04	−0.52**
EDE EC	0.07	0.12	0.25	−0.18	0.13	0.05	0.11	0.18	−0.40	−0.24	−0.30	−0.29
EDE SC	0.21	−0.02	0.09	−0.20	0.21	0.35	0.22	0.31	−0.21	−0.30	−0.25	−0.34
EDI-2 DT	0.14	−0.20	0.23	−0.27	0.02	0.43*	0.24	0.05	−0.27	−0.43*	−0.20	−0.51*
EDI-2 BD	0.30	0.04	0.31	−0.12	−0.36	0.30	0.21	0.33	−0.47*	−0.60*	−0.20	−0.50*
BIQ-VBD	−0.16	−0.14	−0.06	−0.10	0.17	0.08	−0.05	−0.12	0.34	0.40	0.38	0.14
BIQ-NBE	−0.12	−0.03	0.07	−0.05	−0.16	0.27	0.11	0.29	−0.29	−0.47*	−0.25	−0.32
RSE	−0.06	0.08	−0.23	0.18	−0.01	−0.28	−0.15	−0.06	0.49*	0.30	0.36	0.27
PACS	−0.14	−0.03	0.05	−0.06	0.15	0.39	0.22	0.38	−0.29	−0.47*	−0.40	−0.36
STAI pre	0.20	0.03	0.28	0.03	−0.26	−0.30	0.08	−0.28	−**0.42^†^**	−0.08	−0.17	−0.01

†*p* = 0.05, *p < 0.05, **p < 0.01. None of the significant correlations would have survived Bonferroni correction for multiple testing. EDE = eating disorder examination interview (Hilbert & Tuschen-Caffier, 2010; Cooper & Fairburn, 1987), EDE Total,EDE total score; EDE Res, subscale restraint; EDE EC, subscale eating concerns; EDE WC, subscale weight concerns; EDE SC, subscale shape concerns; EDI-2, Eating Disorder Inventory – 2 (Paul & Thiel, 2005); EDI-2-DT, subscale Drive for Thinness; EDI-2-BD, Subscale Body Dissatisfaction; BIQ, Body Image Questionnaire FKB-20 (Clement & Löwe, 1996); BIQ-VBE, subscale vital body dynamics; BIQ-NBE, subscale negative body evaluation; RSE, Rosenberg Self Esteem Scale (Ferring & Filipp, 1996; von Collani & Herzberg, 2003); PACS, Physical Appearance Comparison Scale (Thompson *et al.* 1991; Mölbert *et al.* 2017); STAI, State Trait Anxiety Inventory, State-Form (Laux *et al.* 1981).

### Experiment 2: Perception of a weight and shape matched other person

In Experiment 2, we again used *t* tests to test whether the outcome parameters significantly differed from zero. To test differences between groups and to compare the parameters to Experiment 1, we used mixed ANOVAs with group as a between-subject factor and experiment (1/2) as a within-subject factor. All participants accurately identified the previously memorized body: *Distortion* parameters were not significantly different from zero in any of the tasks and groups [AN: mean 1AFC = −1.48 (s.d. = 3.59), *t*(21) = −1.93, *p* = 0.07, MoA = 0.88 (3.63), *t*(20) = 1.11, *p* = 0.28.; Controls: 1AFC = −0.31 (2.88), *t*(23) = −0.53, *p* = 0.60 MoA = −0.98 (2.76), *t*(23) = −1.73, *p* = 0.10]. Overall, *distortion* parameters were smaller in Experiment 2 than in Experiment 1 [1AFC: *F*_(1,41)_ = 26.64, *p* < 0.001; MoA: *F*_(1,41)_ = 35.21, *p* < 0.001], suggesting that the underestimation observed in Experiment 1 was unlikely due to general perceptual distortions.

Sensitivity to weight change was no longer dependent on the direction of change (weight loss *v*. weight gain) [*F*_(1,40)_ = 1.53, *p* = 0.22]. *Post-hoc t* tests illustrated that this was due to the fact that sensitivity to weight loss was smaller than sensitivity to weight gain as opposed to Experiment 1, indicating a trend in both groups to accept fatter bodies as the correct one [AN: mean *beta_left_ln* = 1.74 (s.d. = 0.58), *beta_right_ln* = 1.59 (0.86); Controls: *beta_left_ln* = 1.97 (0.62), *beta_right_ln* = 1.71 (0.62)].

Similar as in Experiment 1, *desired change* of weight and *desired-v.-actual discrepancy* did not differ significantly from zero in women with AN. As the avatar was matched to the participants’ own weight, this indicates that women with AN found their own current weight most attractive. Again, controls significantly favored a weight loss [AN: mean *DesiredChange* = −2.09 (s.d. = 6.37), *t*(20) = −1.51, *p* = 0.15, *D-v.-A-Discrepancy* = −1.24 (7.48), *t*(20) = −0.75, *p* = 0.46; Controls: *DesiredChange* = −7.16 (5.57), *t*(23) = −6.31, *p* *<* 0.001, *D-v.-A-Discrepancy* = −8.14 (6.07), *t*(23) = −6.56, *p* < 0.001]. The ANOVAs revealed a significant difference to Experiment 1 for *desired change* such that women with AN adjusted a lower attractive weight now [*F*_(1,40)_ = 15.00, *p* < 0.001] and a trend for *desired-v.-actual-discrepancy* to be smaller [*F*_(1,40)_ = 3.57, *p* = 0.066], indicating that the avatar was considered most attractive at a slightly higher weight than the own avatar in Experiment 1.

Taken together, Experiment 2 showed that participants were accurate in memorizing and identifying a body of their own weight and shape. Also, it replicated findings of Experiment 1 in what body weight the participants find most attractive: While women with AN preferred a body at about their own weight, controls preferred a body weighing less than their own current weight.

### Experiment 3: Replication of Experiment 1

*n* = 9 women with AN and *n* = 13 controls participated in Experiment 3. All participants with AN reported ongoing eating disorder symptoms, and BMI still differed significantly between the groups [AN: *M* = 15.87 (s.d. = 2.79], Controls: *M* = 22.14 (2.52), Difference to Exp 1 AN *Z* = −1.007, *p* < 0.32; Controls *Z* = −0.175, *p* < 0.87). All outcome parameters were similar as in Experiment 1 [AN: mean *Distortion_1AFC* = −8.77, s.d. = 8.61, *Distortion_MoA* = −6.69 (8.39), *DesiredChange* = 5.83 (9.44), *D-v.-A-Discrepancy* = 3.93 (9.22), *beta_left_ln* = 0.85 (1.12), *beta_right_ln* = 1.81 (0.35); Controls: *Distortion_1AFC* = −5.78 (5.21), *Distortion_MoA* = −4.36 (7.63), *DesiredChange* = −6.23 (7.66), *D-v.-A-Discrepancy* = −10.39 (6.97), *beta_left_ln* = 1.58 (0.93), *beta_right_ln* = 1.73 (0.69)]. Mixed ANOVAs revealed the same pattern of group differences, but no significant difference to Experiment 1 (all *p* > 0.14), and this was confirmed by nonparametric tests. This suggests that our results from Experiment 1 were robust over time and independent from the presentation device (3D life-size immersive presentation *v*. 2D desktop presentation).

## Discussion

The present study aimed at disentangling perceptual and attitudinal components of BID in AN. To the best of our knowledge, we are the first to use biometric self-avatars in virtual reality to investigate body image in AN. Our methods allowed us to realistically manipulate body weight of personalized avatars and to investigate perception of other bodies in a well-controlled way by changing the identity of the avatar while keeping the underlying body shape identical. Also, we minimized demand characteristics by using psychophysical experiments and by implementing an outside-treatment-setting for our study. According to our observations, women with AN neither see their own body nor other weight-matched persons differently than controls, but they evaluate them differently in terms of what weight is desirable. Hence, while visual perception of their body is normal, attitudinal components of body representation are strongly disturbed. In the clinical context, our findings suggest that patients with AN need support in changing their desired weight and in feeling positive about a normal weight body.

In this study, we investigated a severely affected patient sample. Importantly, all participants with AN were already in treatment and on their way to partial remission, as illustrated by their EDE scores being lower than in other samples of patients with AN (Hilbert *et al.* 2004). However, all women with AN reported anorexia-typical cognitions and behavior as possible in the treatment setting. The control participants were representative for their age, as illustrated by their scores in questionnaires and the EDE interview (Clement & Löwe, 1996; Ferring & Filipp, 1996; von Collani & Herzberg, 2003; Paul & Thiel, 2005; Hilbert & Tuschen-Caffier, 2010).

The manipulation check confirmed that, as expected, participants identified more with their photo-realistic self-avatar than with the weight and shape matched avatar in Experiment 2. The patients’ overall lower identification with the avatars can be explained in the context of their eating disorder symptoms: Women with AN reported high body dissatisfaction as well as low experience of vital body dynamics for their own body. Additionally, they were more anxious before the experimental session. Hence, their overall lower identification might express a general ‘distance’ that participants with AN felt toward their body and also for their avatar.

Body representation has longtime been conceptualized as a hierarchical construct with different components (Berlucchi & Aglioti, 2010; de Vignemont, 2010). As there is no clear evidence for any such distinction (de Vignemont, 2010), a dimensional model has recently been developed (Longo *et al.* 2010; Longo, 2015, 2016). In this notion, body representation is a conglomerate of multiple body representations that can be characterized in terms of how explicit *v*. implicit they are and in how much they are perceptual *v*. conceptual. The body representations are informed by different senses and modalities, such as vision, proprioception or even social comparison and can be integrated into higher-level representations. A benefit of this framework is that it supports a distinction between perceptual and conceptual representations while at the same time considering mutual interactions. From our experimental tasks, we were able to derive different measures of explicit visual body perception. If distorted visual perception or low BMI were the driving factors behind overestimation, we would have expected to observe overestimation in all experiments, whereas overestimation in Experiment 1 only would have suggested demand characteristics or other self-referring processes as driving factors. Interestingly, irrespective of group, participants tended to underestimate their weight in Experiment 1, and there was a trend that this was even more pronounced in women with AN than in controls (cf. Table 2 and Fig. 2). In line with a previous study (Øverås *et al.* 2014), we observed more accurate estimations in Experiment 2, suggesting that mis-estimation of the own size was linked to own identity.

Similarly, none of the sensitivity parameters showed a group effect indicative of a poorer performance in women with AN, and Experiment 3 suggested that this finding is robust. However, there was a trend in both groups to accept thinner avatars as corresponding to the own body, while for the memorized other person in Experiment 2, fatter bodies were more readily accepted as correct. A possible explanation for the underestimation and higher acceptance of thinner bodies as own in Experiment 1 is that participants’ memories of their own bodies were influenced by a self-serving bias that is that participants remembered themselves closer to their desired weight (Aars & Jacobsen, 2016). However, this explanation would be discrepant with studies showing that people with an eating disorder tend to focus their attention on body parts perceived as non-attractive when they see their own body, whereas they focus on attractive body parts in other people (von Wietersheim *et al.* 2012; Tuschen-Caffier *et al.* 2015). Alternatively, it is possible that although participants remembered their body accurately, they additionally based their judgments on conceptual representations such as ‘this body is lean’ or ‘I am thin’ (Smeets *et al.* 2009). Overall, our observations suggest that body size estimation in women with AN is not generally characterized through a deficit in visual weight representation. However, given that we observe cognitive–attitudinal influences even on allegedly perceptual parameters such as sensitivity (Gardner & Moncrieff, 1988), our observations also emphasize how challenging it is to isolate specific representations of the body through experimental tasks.

A further strategy to investigate whether visual perceptual distortions might underlie BID in AN was to analyze whether distortion or sensitivity correlate with eating disorder symptoms or body dissatisfaction. We observed no significant correlation between the distortion or sensitivity parameters of Experiment 1 with eating disorder symptoms and body dissatisfaction. Further, unlike a previous study, we did not observe that anxiety is associated with overestimation of body size (Øverås *et al.* 2014). Interestingly, we also observed no correlation with BMI, suggesting that in our paradigm, overestimation was neither associated with eating disorder symptoms nor a secondary effect to low BMI (Cornelissen *et al.* 2013, 2015). Several differences between previous studies and the present setup could account for these discrepancies; we used for example a different stimulus presentation method and task instruction.

In line with existing literature (Cash & Deagle, 1997; Mohr *et al.* 2010; Sala *et al.* 2012), we observed a consistent preference of women with AN for severely underweight bodies. While controls’ desire for a lower weight can be interpreted as common desire for a slender healthy weight body (Aars & Jacobsen, 2016), the desired weight of women with AN is concerning. Although women with AN had committed themselves to clinical treatment, had expressed insight in their current weight status when estimating their size, and often adjusted a *desired change* in the direction of weight gain, only five women with AN actually adjusted a desired body weighing more than themselves in Experiment 1 (Fig. 2*c*). Notably, more than half (52%) of women with AN desired a body that would have been in the weight range of extreme AN (i.e. BMI below 15), although all women with AN would have been able to adjust the body outside that weight range. Our observations show that although women with AN know about their underweight, they have large difficulties in internalizing a normal weight as goal and in stopping to ‘like’ their current underweight.

The present study also has limitations: First, although our paradigms allow for strong conclusions on the role of visual perception for body size estimation, we have not examined other sensory modalities. As body representation is a very complex and broad construct (Longo *et al.* 2010; Longo, 2016), it is possible that our paradigm has overlooked nonvisual perceptual disturbances which might be involved in the feeling of being too fat that women with AN often report. Second, while the statistical shape model used in this study is one of the most realistic to date, it was built to represent the shape of a normal weight population and may not perfectly characterize variations in weight at the extreme end of the spectrum. Further, we see a limitation in that we varied participants’ bodies in a range of ±20% of their own weight instead of a fixed weight range, e.g. from underweight to normal weight. Although this prevented biases due to Weber's law (Cornelissen *et al.* 2016), it also led to different absolute weight spectrums and limited the range in which participants could adjust their desired body.

Our study contributes to a better understanding of the nature and mechanisms of BID in AN and it has direct implications for the treatment of AN. Our observations contradict the widespread assumption that patients with AN have a perceptual distortion in the sense that they cannot accurately see their own size or perform generally bad in estimating body sizes. Rather, we find evidence that attitudinal components of body image are distorted in AN, as affected individuals consider underweight bodies as desirable and attractive. It remains open whether other sensory modalities contribute to this attitudinal disturbance. According to our observations, interventions should aim at helping patients with AN to change their desired weight and to accept their body in healthy weight. Further studies are needed to explore in more detail at what level of body representation interventions are most promising.

## Supplementary material

For supplementary material accompanying this paper visit http://dx.doi.org/10.1017/S0033291717002008. click here to view supplementary material
