# Supplementary material for: Assessing body image in anorexia nervosa using biometric self-avatars in virtual reality: Attitudinal components rather than visual body size estimation are distorted
Source: Psychol Med. 2017 Jul 26;48(4):642–53. doi: 10.1017/S0033291717002008 (PMC5964466; doi:10.1017/S0033291717002008)
Supplement: Supplementary file 1 [file S0033291717002008sup001.docx]

***Supplementary material to method section of the manuscript “Assessing body image in anorexia nervosa using biometric self-avatars in virtual reality: Attitudinal components rather than visual body size estimation are distorted”***

## Body scan and stimulus image generation

As described in the main text, we collected body scans using a full body 3D scanning system (3dMD, Atlanta, GA). The scanning system uses speckle projectors which project textured light patterns on the body, 22 stereo units composed of two black and white cameras observing the speckle pattern for recording the body geometry, and a 5-megapixel colour camera capturing the body texture. The system has a spatial resolution of approximately 1 mm. In order to get accurate representations of the participants’ body shapes, participants dressed in a standardized set of tightly fitting short grey pants and a grey top and if they had long hair, dressed their hair to a bun. We took three body scans in T-pose, A-pose and neutral pose, resulting in three high-polygon meshes and three RGB images for texture generation.

These meshes were then co-registered to a statistical model of body shape that parametrizes individual shape (Anguelov *et al.* 2005; Hirshberg *et al.* 2012). The statistical body model consists of a template mesh that can be deformed in shape and pose in order to fit a 3D-scan. The shape component of the body model was learned from 2,094 female bodies in the CAESAR dataset (Robinette *et al.* 1999) by applying principal component analysis on the triangle deformations in the observed meshes after removing deformation due to pose. This allowed to model body shape variation in a subspace, *U*, spanned by the first 300 principal components, where the body shape of an individual, *S_j_*, is described as a vector of 300 linear coefficients, *β_j_*, that approximate the shape deformation as *S_j_* = *Uβ_j_* + *μ*, where *μ* is the mean shape deformation in the female population. The pose component of the body model compactly describes deformations due to body part rotations and is trained from approximately 1,200 3D-scans of people in different poses. The registration process consists of the identification of pose and shape parameters that transform the template mesh into the scan by minimizing the distance between template mesh and scan. Once the scan is registered, a texture map is computed for the participant’s model based on the pixels from the 22 RGB calibrated images. The final texture map was computed using the median pixels of the three textures. This texture map was later post-processed in Adobe Photoshop (CS6, 13.0.1) to conceal small artefacts and to standardize brightness and the colors of the textures across participants.

In order to generate the different BMI versions of each avatar, a linear regressor X was learned between anthropomorphic measurements *A* = [weight, height, arm length, inseam] and the shape identity component *β* for the whole CAESAR dataset, so that the difference ||(*A*|1)*X* – *β*|| is minimized*.* This defines a linear relation between shape and measurements for each participant and allowed us to modify *β* in a way that produces intended changes in the anthropomorphic measurements. Given each participant’s weight *w*, height *h*, and registration, nine avatars were generated with varying BMIs $\left( 1+\frac{\Delta BMI}{100} \right)\frac{w}{h^{2}}$, where *∆BMI* = {0, ±5%, ±10%, ±15%, ±20%}. Changing the BMI was achieved by applying a change in the shape vector, so that $\Delta\beta=\left[ \frac{\Delta BMI}{100}w, 0, 0, 0 \right]\cdot X$ (i.e. changing the weight equally to the desired proportional change in BMI, while keeping the other measurements – height, arm length, inseam – constant (see Piryankova et al., 2014 for a more mathematical description). An example of these shape deformations applied to an average body is provided at <http://bodyvisualizer.com/> (Max Planck Institute for Intelligent Systems, Perceiving Systems, 2011). To prevent possible effects due to individual pose, pose parameters of all avatars were standardized across participants to an A-pose. The pose parameter vector was calculated as the average pose parameter vector of all registered scans in the A-pose from the CAESAR dataset.

The nine body shapes were then combined in Autodesk 3ds Max 2015 to a single avatar with morph channels such that it was possible to morph between the meshes in steps of 0.05% of the participant’s actual BMI. Finally, the body was horizontally flipped in order to enable a second-person (mirror) perspective as opposed to a third-person (photo) perspective on the body.

To generate the artificial other person‘s avatar for Experiment 2, we used the participant’s body meshes, but presented them not with the participant’s texture map, but with a standard texture map. This standard texture was generated by combining the median pixels of 1,200 scans used for the pose model (around 50 different women), so that it represents an average person. That way, we obtained an avatar that was completely matched to the participant in terms of height, weight and body shape, but due to different color information appeared like having another identity.

## Experimental technical setup

We implemented the experimental setups for both the One-Alternative Forced Choice and the Method of Adjustment Task in Unity 3D (Version 4.6.3f1, Unity Technologies). We placed the participant’s mirror-inverted avatar in an empty virtual room at a distance of 2 m from the participant, i.e. 1 m from the screen (Fig 1, left). In Experiment 1 and 2, participants stood at 1 m distance from the screen, so that for them the scene looked like facing themselves in a mirror (see Figure 1). The scene was presented a flat, large-screen immersive display on which the stimuli were projected using a Christie SX+ stereoscopic video projector (1400 x 1050 native pixel resolution). The projected area covered 2.16 m width × 1.62 m height (94.4° x 74° of visual angle) with a floor offset of 0.265 m. The stereoscopic projection was generated using an average interocular distance of 6.5 cm (Willemsen *et al.* 2008). In order to see the scene stereoscopically, participants wore a pair of shutter glasses (nVidia 3D Vision Pro). The glasses had a field of view of 103° × 62°, corresponding to an area of 2.52 m × 1.2 m of the display. The display was connected to a motion tracking system (ART, SMARTTRACK). Although the avatar was a three dimensional object, perspective was locked to frontal view, so that the body could only be seen from the front, but not from the side. Despite limited interaction, the setup scored high on central dimensions of virtuality as described in Milgram and Kishino (1994). Visual angle of the whole avatar was approx. 29.5-32° (torso 8-12°), although it depended on participants’ size and the morph width. However, participants were allowed to look around freely.


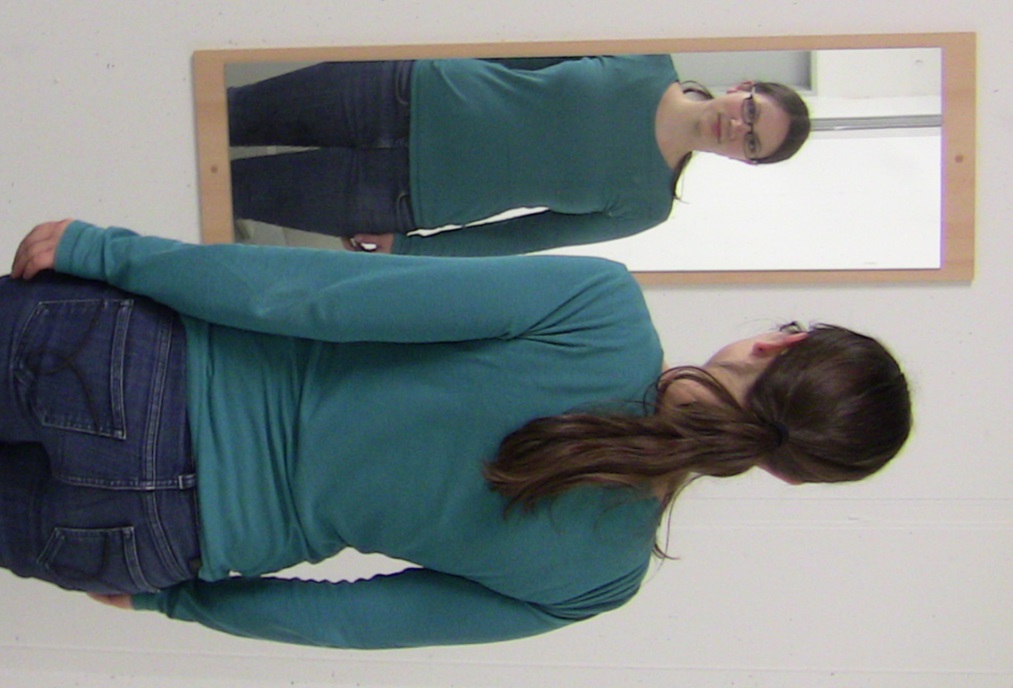


Figure S1. A+B Illustration of life-size stimulus presentation in Experiment 1 and 3, mimicking the situation of looking at oneself in a mirror. C Screenshot of avatar presentation in Experiment 3

For Experiment 3, we exported the Unity scene for desktop use. Presentation devices were not standardized, but ordinary desktop monitors were used that were placed so that the avatar was shown approximately at participants’ eye height. The scene was displayed in full screen mode. Responses were given using the left and right buttons of a computer mouse. During the task, participants were seated, resulting in a weaker mirror illusion due to incongruent size and pose between participants and their avatar.

**C**

**B**

## Experimental procedure

As described in the main document, the experimental session (2) consisted of two blocks, the first containing Experiment 1 and the second containing Experiment 2. In Experiment 1, body size of the avatar with own photorealistic texture was estimated. In Experiment 2, participants estimated the size of a memorized avatar with another identity. To the participants, this avatar was introduced as “another person” without any further explanation. However, the avatar used in Experiment 2 had the identical body shape as the participant, but a standard identity (texture). Experiment 3 contained the same psychophysical tasks and the same avatar as Experiment 1, but the body stimuli were presented on a desktop monitor.

In all experiments, participants completed three tasks: A One-Alternative Forced Choice Task (1AFC) and two Method of Adjustment Tasks (MoA), one referring to the current and one referring to ideal body size. Prior to Experiment 1 and 3, participants were informed that based on the body scan, a set of personalized bodies had been generated that could either represent exactly their body or be gradually shrunk respectively blown up versions of their body. Prior to Experiment 2, participants were informed that they now had to memorize another body. Afterwards, they would be shown correct, blown up and shrunk versions of the body and would have to identify the correct body. Participants were presented with the template body for 2 minutes before the 1AFC task and again for 1 minute before the MoA task.

In the 1AFC task participants were shown their avatar for 2 seconds and afterwards a blue screen with a statement appeared. In Experiment 1 and 3, the statement was “That was my body. (Yes/No)” and in Experiment 2, it was modified to “That was the correct body (Yes/No)”. The wording was chosen as emotionally neutral alternative to frequently used thinner/fatter judgments. No time limit was given for the answer, but participants were instructed to rely on their gut feeling for the answers and to not cogitate much. Answers were given through pressing left and right buttons on a joystick pad that participants held in their hands. As soon as the participant had answered, the next trial started. Avatars were morphed to ± 0%, 5%, 10%, 15% and 20% weight, and each of these nine body shapes was presented 20 times, resulting in a total of 180 trials. The order of the trials was randomized block-wise such that each of the nine different bodies was presented before being presented again. After every 45 trials, participants could take a break if needed.

In the MoA task, participants were shown the avatar with no time limit, and had to adjust it to their current respectively ideal body size. At the beginning of each trial a blue screen with the instruction appeared for 2 seconds, afterwards, the avatar appeared. In Experiment 1 and 3, the instruction was “Please adjust the body until it matches your CURRENT body! (Less / More)”, in Experiment 3 it was modified to “Please adjust the body until it matches THE CORRECT body! (Less / More)”. Participants could again use the left and right button of a joystick pad to do the adjustment, and started the next trial using another button on top of the joystick pad. During the procedure, no time limit was given, and participants were allowed to go back and forth. Each of the nine weight steps used in the 1AFC task was randomly presented once as a start body, resulting in nine trials. Linear morphing was possible continuously in steps of 0.05% of the participant’s BMI, again in the range of ±20%. Finally, for the MoA ideal body, the MoA procedure was repeated, with instructions modified to “Please adjust the body until it matches the CORRECT body” respectively to “Please adjust the body so that it is as ATTRACTIVE as possible”.

## Outcome measures

From the different experimental tasks, we extracted for all experiments 1) the degree of inaccuracy/distortion of the estimated body size as compared to participants’ actual weight at the time of the experiment (1AFC and MoA) 2) the sensitivity to weight changes when avatars were morphed to lose and gain weight (1AFC) 3) the desired weight change (MoA) and 4) the discrepancy between desired and actual weight (MoA).


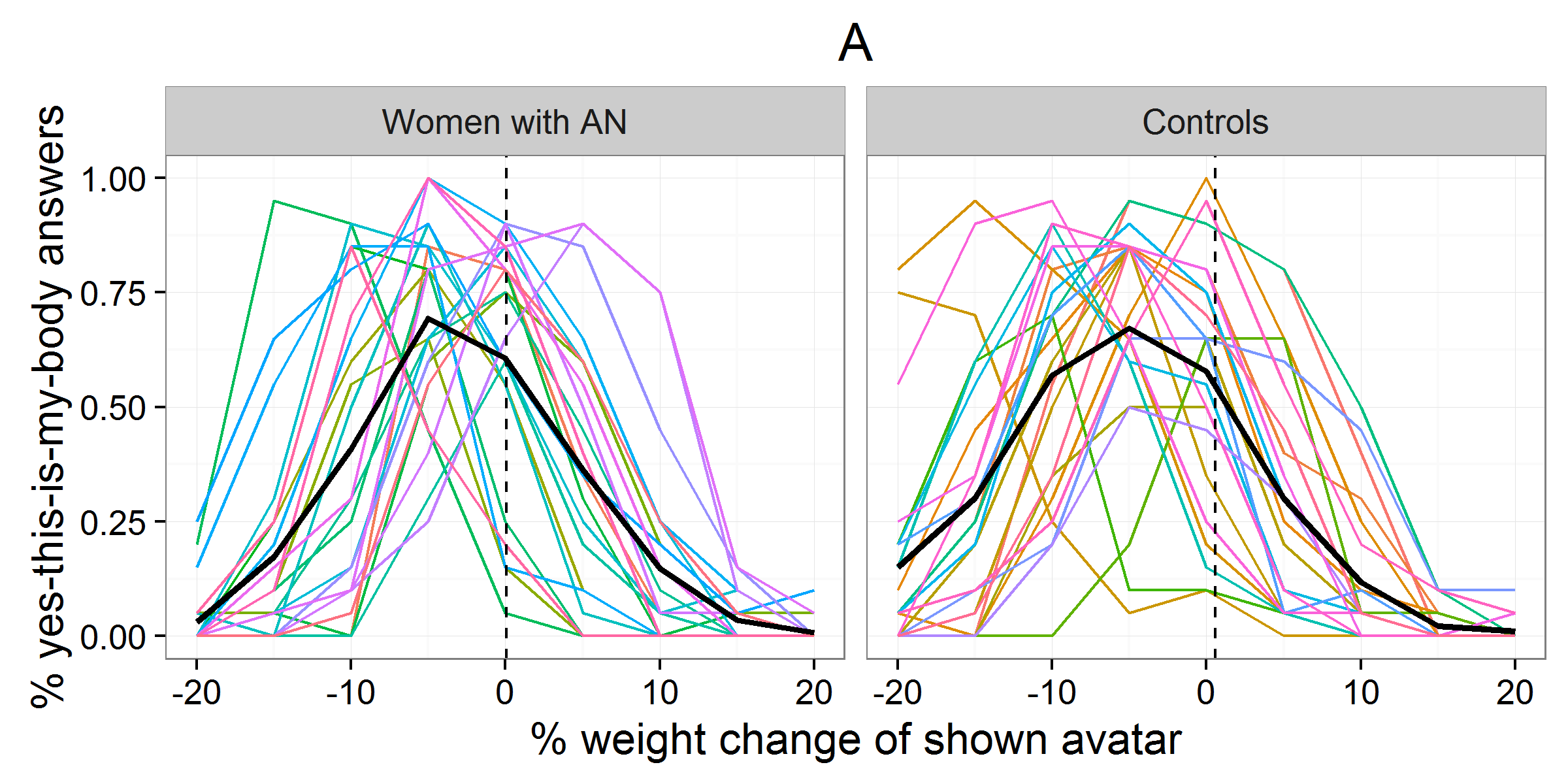


Figure S2. Proportion of yes-this-is-my-body answers depending on the BMI change of the shown avatar for the single participants (colored lines) and average responses (black lines) from the 1AFC task in Exp. 1. The dashed vertical line indicates accurate performance as compared to average weight at the time of the experiment. For the data analysis, we extracted for each participant the peak of the answer distribution as well as the steepness of the answer distribution left and right from the peak.

To quantify the degree of distortion, we computed the over- or underestimation

relative to actual individual body weight. Specifically, the formula we used was *distortion* = *(estimated weight/actual weight at experiment)*100 - 100*. Negative values reflect an underestimation of weight, while positive reflect an overestimation in percent of the participants’ actual weight. In the 1AFC task, the weight of the body shape with the highest proportion of yes answers to the statement “This is my body”, that is the mode of the distribution of yes-answers over the weight steps was used as the “estimated weight” for the calculation of body size estimation distortion (cf. figure S2). In some cases, two adjacent body shapes had the same amount of yes-answers and then, their average weight was used as mode. In the MoA task, we used the average weight of the adjusted avatars in the nine trials as “estimated weight” to calculate *distortion*. Trials were excluded from the analysis if the estimated body weight was larger than 2 standard deviations from the mean response of the remaining trials per participant and condition (current, ideal), since some participants reported that they had accidentally confirmed the adjusted size before they were finished with their adjustments. This affected 3.7% of all trials in Experiment 1 and 5.4% of all trials in Experiment 2. Due to a technical error discovered after data collection was finished, linear morphing in the MoA task did not always occur between 15% and 5% of participants’ weight when decreasing the avatar’s weight. We therefore investigated the time course of adjusting the avatar’s weight in each trial. Only 3.8% of the final responses (4.5 % in experiment 2) were given under influence of this displaying error. In experiment 1, 30% of the affected trials were from the same person and very consistent. In experiment 2, affected trials were distributed over all participants. Since participants had no time limit for the adjustments, were explicitly instructed to go back and forth until they felt they couldn’t do it more accurate, and the time courses also reflected that they followed our instruction, we decided not to exclude these trials as this might have biased the results for people that responded in this range. Sanity checks confirmed that excluding the affected trials did not affect significance levels of any statistical analysis.

As a measure of *sensitivity*, we analyzed the steepness of the curve on both sides of the mode for each participant and experiment. To parametrize the steepness, we fitted cumulative Weibull functions according to Wichmann and Hill (2001) to both sides of the respective answer distributions. Alpha (position of the psychometric function along the x-axis), beta (the slope of the psychometric function), and lambda (the peak of the psychometric function) were free to vary. Gamma (flooring performance) was fixed to zero. In Experiment 1, good fits were yielded with *R²* = 0.997 (Min = 0.96, Max = 1.0) on the left side and *R²* = 0.993 (Min = 0.95, Max 1.0) on the right side of the answer distributions. The fitting did not succeed for the left side for one woman with AN and for two control participants due to lack of data. Similarly, in Experiment 2, fits were good with *R²* = 0.996 (Min = 0.933, Max = 1.0) and *R²* = 0.992 (Min = 0.934, Max = 1.0) on the right side.

*Desired change* of weight was defined as the required percent weight change to make the body the desired/most attractive body as compared to the estimated body. To this end, we computed similar to the procedure for *distortion* how many percent of actual weight the participant desired to lose or gain to make the body the desired one as compared to what she estimated as her current body. Again, the average adjusted “ideal” body weight of the nine trials was used to calculate the desired weight and the average adjusted “current” body from the MoA task was used as “estimated weight”. The desired change of weight as computed here can also be interpreted as body dissatisfaction with absolute values reflecting the degree of body dissatisfaction and polarity reflecting whether the participant whishes an upward or downward change.

In order to also obtain a measure of how far the adjusted desired body was off from the actual weight, we additionally computed the *actual-vs-desired* *discrepancy* as the percent weight discrepancy between the desired body (or most attractive body) and the actual body weight at the experiment*.* As opposed to the parameter *desired change*, *actual-vs-desired discrepancy* does not express the subjective, explicit wish and direction for weight change, but provides a more objective measure for what weight the participant finds most attractive.

## Statistical Analysis

All statistical analyses were conducted using IBM SPSS Statistics 24. As a general check, we first analyzed group differences in age, height, weight, body mass index, questionnaire measures of body dissatisfaction, self-esteem, comparison habits with regard to outer appearance and symptoms of eating disorders using t-tests for normally distributed data and U-tests for non-normally distributed data. We also analyzed group differences in anxiety before the experiment and anxiety in- or decrease throughout the experiment. As a manipulation check, we calculated a mixed analysis of variance (ANOVA) with between-factor group (AN versus Controls) and within-factor experiment (1 versus 2) on similarity ratings of the avatars to check whether participants in both groups really experienced the “other” body as less similar to themselves than the self-avatar with own texture.

We conducted one-sample t-tests to test whether the *distortion* parameters were significantly different from zero, i.e. whether the participants were significantly inaccurate in their estimates. To examine group differences in *distortion*, we calculated univariate ANOVAs with between-subjects factor group (AN versus Controls) *distortion* parameters from both 1AFC and MoA. The *sensitivity* to weight change parameters beta were analyzed together in one mixed ANOVA with between-subjects factor group (AN versus Controls) and side of the peak (left versus right) as additional within-subject factor so that we could assess whether participants of the two groups were equally sensitive to weight changes in the losing and gaining direction. Since sensitivity parameters beta were not normally distributed but significantly right skewed, they were log-transformed prior to the analysis. *Desired change* of weight and *desired-vs-actual discrepancy* were analyzed similarly to the *distortion* parameters: First, one-sample t-tests were used to determine whether *desired change* of weight and *desired-vs-actual discrepancy* differed significantly from zero, and then we used univariate ANOVAs to compare the values between the two groups.

Next, we explored separately for women with AN and controls correlations between the outcome parameters for the self-avatar with own texture and body mass index, body dissatisfaction, self-esteem, comparison habits with regard to outer appearance and symptoms of eating disorders or anxiety before the experiment.

Finally, to assess whether the same pattern of results would reveal in Experiment 2 with the other person’s texture, we repeated the above ANOVAs with experiment (Experiment 1 versus Experiment 2) as an additional within-subjects factor. Experiment 3 was analyzed analogously and parameters were compared to Experiment 1 using the ANOVA with group (AN versus Controls) as between-subjects factor and experiment (Experiment 1 and 3) as within-subjects factor. In average participants’ weight did not change significantly in any of the groups between Experiment 1 and 3 (AN: mean change = +.0.96 BMI units; *Z* = –1.01, *p* = .31; Controls: mean change = +0.07 BMI units; *Z* = –0.18, *p* = .86;). To control for individual weight changes, all outcomes were calculated with respect to current weight.

## References

**Anguelov D, Srinivasan P, Koller D, Thrun S, Rodgers J, Davis J** (2005). Scape: shape completion and animation of people. *ACM Transactions on Graphics*, 408–416.

**Hirshberg D, Loper M, Rachlin E, Black MJ** (2012). Coregistration: Simultaneous alignment and modeling of articulated 3D shape. *Proceedings of the European Conference on Computer Vision (ECCV). Lecture Notes in Computer Science* **7577**, 242–255.

**Milgram P, Kishino F** (1994). A taxonomy of mixed reality visual displays. *IEICE Transactions on Information Systems* **E77**–**D**, 1321–29.

**Piryankova I V, Stefanucci JK, Romero J, De La Rosa S, Black MJ, Mohler BJ** (2014). Can I Recognize My Body’s Weight? The Influence of Shape and Texture on the Perception of Self. *ACM Transactions on Applied Perception* **11**, 1–18.

**Robinette KM, Daanen H, Paquet E** (1999). The CAESAR project: a 3-D surface anthropometry survey. In *Second International Conference on 3-D Digital Imaging and Modeling*, pp380–386. IEEE Comput. Soc.

**Wichmann AF, Hill NJ** (2001). The psychometric function: I. Fitting, sampling, and goodness of fit. *Perception & psychophysics* **63**, 1293–313.

**Willemsen P, Gooch AA, Thompson WB, Creem-Regehr SH** (2008). Effects of Stereo Viewing Conditions on Distance Perception in Virtual Environments. *Presence: Teleoperators and Virtual Environments* **17**, 91–101.
